# Peptains block retinal ganglion cell death in animal models of ocular hypertension: implications for neuroprotection in glaucoma

**DOI:** 10.1038/s41419-022-05407-2

**Published:** 2022-11-15

**Authors:** Mi-Hyun Nam, Dorota L. Stankowska, Gretchen A. Johnson, Rooban B. Nahomi, Mina B. Pantcheva, Ram H. Nagaraj

**Affiliations:** 1 Sue Anschutz-Rodgers Eye Center and Department of Ophthalmology, School of Medicine, Aurora, CO USA; 2 Department of Pharmacology and Neuroscience, North Texas Eye Research Institute, UNT Health Science Center, Fort Worth, TX 76107 USA; 3 Department of Pharmaceutical Sciences, Skaggs School of Pharmacy and Pharmaceutical Sciences, University of Colorado, Anschutz Medical Campus, Aurora, CO 80045 USA; 4 Present Address: Essent Biologics, Centennial, CO 80111 USA

**Keywords:** Neurodegeneration, Neurodegenerative diseases

## Abstract

Ocular hypertension is a significant risk factor for vision loss in glaucoma due to the death of retinal ganglion cells (RGCs). This study investigated the effects of the antiapoptotic peptides peptain-1 and peptain-3a on RGC death in vitro in rat primary RGCs and in mouse models of ocular hypertension. Apoptosis was induced in primary rat RGCs by trophic factor deprivation for 48 h in the presence or absence of peptains. The effects of intravitreally injected peptains on RGC death were investigated in mice subjected to retinal ischemic/reperfusion (I/R) injury and elevated intraocular pressure (IOP). I/R injury was induced in mice by elevating the IOP to 120 mm Hg for 1 h, followed by rapid reperfusion. Ocular hypertension was induced in mice by injecting microbeads (MB) or silicone oil (SO) into the anterior chamber of the eye. Retinal flatmounts were immunostained with RGC and activated glial markers. Effects on anterograde axonal transport were determined by intravitreal injection of cholera toxin-B. Peptain-1 and peptain-3a inhibited neurotrophic factor deprivation-mediated RGC apoptosis by 29% and 35%, respectively. I/R injury caused 52% RGC loss, but peptain-1 and peptain-3a restricted RGC loss to 13% and 16%, respectively. MB and SO injections resulted in 31% and 36% loss in RGCs following 6 weeks and 4 weeks of IOP elevation, respectively. Peptain-1 and peptain-3a inhibited RGC death; the loss was only 4% and 12% in MB-injected eyes and 16% and 15% in SO-injected eyes, respectively. Anterograde transport was defective in eyes with ocular hypertension, but this defect was substantially ameliorated in peptain-injected eyes. Peptains suppressed ocular hypertension-mediated retinal glial activation. In summary, our results showed that peptains block RGC somal and axonal damage and neuroinflammation in animal models of glaucoma. We propose that peptains have the potential to be developed as therapeutics against neurodegeneration in glaucoma.

## Introduction

Glaucoma is a progressive optic neuropathy and is a major cause of irreversible blindness worldwide. Among several types of glaucoma, primary open-angle glaucoma is the dominant type [1]. Approximately 80 million people worldwide are afflicted with glaucoma; this number is projected to grow significantly in the coming years; it is estimated that worldwide there will be ~110 million people suffering from glaucoma by 2040 [2].

The loss of vision in glaucoma occurs due to the death of retinal ganglion cells (RGCs) [3] accompanied by optic disc neuropathy. Elevated intraocular pressure (IOP) is a major risk factor for RGC death in glaucoma [4]. Numerous studies have investigated causes of RGC death and have identified specific organelle defects or abnormalities in biochemical mechanisms, including elevated levels of endothelins [5], mitochondrial dysfunction [6], oxidative [7] and nitrosative [8] stress, neuroinflammation [9], ischemic hypoxia [10], and vascular deficits [11]. The primary mode of treatment for glaucoma is to lower IOP. This is achieved by administration of topical medications, laser treatment, or intraocular surgeries. Despite these interventional methods, some patients’ vision further deteriorates due to the continued death of RGCs [12]. Numerous studies have tested small molecules and biologics against RGC death in experimental animal models of glaucoma [13–16]. Several of them have shown promising results. In addition, recent studies have also indicated promising neuroprotective effects of gene therapies in animal models of glaucoma [17–20]. However, despite these advances, none have received US Food and Drug Administration approval for glaucoma treatment.

Small heat shock proteins (sHsps) are a family of proteins consisting of 11 members [21, 22]. They are anti-apoptotic proteins with chaperone functions. Through their chaperone activity, they bind to partially unfolded proteins and prevent their denaturation in an ATP-independent manner [23]. With the aid of other proteins, the chaperoned client’s biological activity is restored, and it is then released [24]. The anti-apoptotic activity of sHsps lies in their ability to block cytochrome C release and TRAIL-induced caspase-3 activation and to inhibit Bax translocation to the mitochondrial membrane [25–28].

HspB5 (αB-crystallin) is a member of the small heat shock protein family. It is expressed in several retinal cell types, including RGCs. HspB5 levels are reduced in human glaucomatous retinas and in an animal model of glaucoma [29, 30]. Several studies have shown that the delivery of HspB5 into the vitreous humor protects RGCs in experimental models of glaucoma [30–33]. A short peptide within the α-crystallin domain of HspB5 (peptain-1), D^73^RFSVNLDVKHFSPEELKVK^92^, was shown to possess both the chaperone [34, 35] and antiapoptotic activities [36, 37] of the full protein [25]. In our previous studies, we demonstrated that systemic delivery of this peptide inhibits lens epithelial cell apoptosis and cataract development in rats [37] and RGC death in animal models of glaucoma [29]. We have also demonstrated that peptain-1 restores retinal mitochondrial cytochrome C oxidase subunit 6B2 levels [29]. We tested peptain-1 through systemic administration in these studies, which served as proof-of-concept studies and not as a prelude to possible therapies for cataracts and glaucoma.

Peptain-3, G^72^HFSVLLDVKHFSPEEIAVK^91^, is derived from the α-crystallin core domain of HspB6 (Hsp20). We have previously shown that this peptide exhibits chaperone and anti-apoptotic activities [38]. We introduced acetylation at K81 (peptain-3a, G^72^HFSVLLDVK(acetyl)HFSPEEIAVK^91^) to increase resistance to proteolytic digestion when injected intravitreally. Our results showed the acetyl peptide exhibited robust chaperone activity (Supplementary Fig. 1). This peptide has not been tested for neuroprotective properties until now.

Topical or intravitreal administration are the preferred routes for drug delivery to treat ocular diseases since they limit undesirable systemic effects. Examples of intravitreal administration include the injection of vascular endothelial growth factor trapping molecules to treat neovascular age-related macular degeneration [39] and diabetic macular edema [40]. Such intravitreal treatments have helped to save vision in millions of people. Based on these successes, we reasoned that intravitreal injection, and not the systemic delivery that we used in our previous study on peptain-1 [29], would be the better avenue to deliver peptains into the retina for neuroprotection in glaucoma. In this study, we report the impacts of intravitreally delivered peptains on RGC survival following ischemia/reperfusion (I/R) injury and in two models of ocular hypertension in mice.

## Results

### Peptains protected primary rat RGCs against neurotrophic factor deprivation

**Fig. 1:**
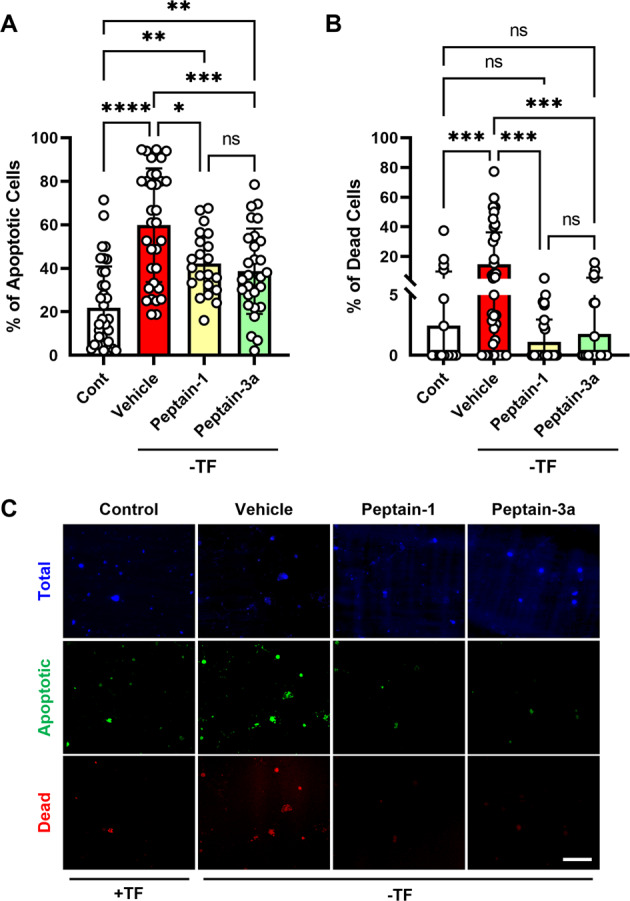
Peptains protect RGCs from apoptosis due to neurotrophic factor deprivation. Rat RGCs were isolated, cultured, and subjected to neurotrophic factor deprivation in the presence or absence of peptains. Percentages of apoptotic (**A**) and dead (**B**) RGCs in the absence of trophic factors (TF) after 48 h of incubation. Representative images of total (blue), apoptotic (green), and dead (red) RGCs after 48 h in the presence or absence of TF (**C**). The bars represent the mean ± SD of three independent experiments, and each data point represents the percentage of apoptotic/dead cell counts from a specific region in the image. *p < 0.05, **p < 0.01, ***p < 0.001, ****p < 0.0001, ns = not significant. Scale bar = 100 µm.

Our previous study showed that peptain-1 is permeable to RGCs and inhibits RGC apoptosis induced by hypoxic stress [29]. Based on that study, we evaluated the ability of peptain-1 and peptain-3a to prevent apoptosis caused by neurotrophic factor deprivation in rat primary RGCs in vitro. Since a scrambled peptide did not show protective effects against RGC death in an earlier study [29], we did not include a similar control peptide in this study. The observation that the anti-apoptotic activity of an sHsp (HspB4) is directly related to its chaperone activity [41] suggested that the chaperone activity of sHsp-derived peptides can be used as a measure of their antiapoptotic activity. We observed that the chaperone activity of peptain-3a was 10-fold better than that of peptain-1 (Fig. S1). Therefore, we treated cells with a 10-fold lower concentration of peptain-3a than peptain-1 (1.25 µg/mL of peptain-3a vs. 12.5 µg/mL peptain-1). When RGCs were cultured in the absence of trophic factors (TF), there was an increase in the number of apoptotic (by 2.7-fold) and dead cells (by 6-fold) over cells cultured in the presence of TF. Treatment with peptain-1 and peptain-3a significantly reduced the apoptotic cell numbers by 29% (*p* < 0.01) and 35%, respectively (*p* < 0.0001), and dead cells by 92% (*p* < 0.01) and 87% (*p* < 0.0001) when compared to cells cultured in the absence of TF (Fig. 1A–C). Together, these results suggested that peptains were effective in protecting RGCs under stressful apoptosis-promoting conditions of TF deprivation. Furthermore, peptains did not induce cell death or apoptosis in normal growth conditions in the presence of TF (Fig. S2A, B). Under the basal conditions, there were a few apoptotic cells; those numbers were unaffected by the peptain treatment. However, the dead cell counts were significantly lower by the peptain-1 (*p* < 0.05) and peptain-3a (*p* < 0.01) treatment relative to untreated controls, which suggested that peptains were able to protect RGCs from spontaneous cell death in cultured RGCs.

### Peptains inhibit RGC loss from I/R injury

**Fig. 2:**
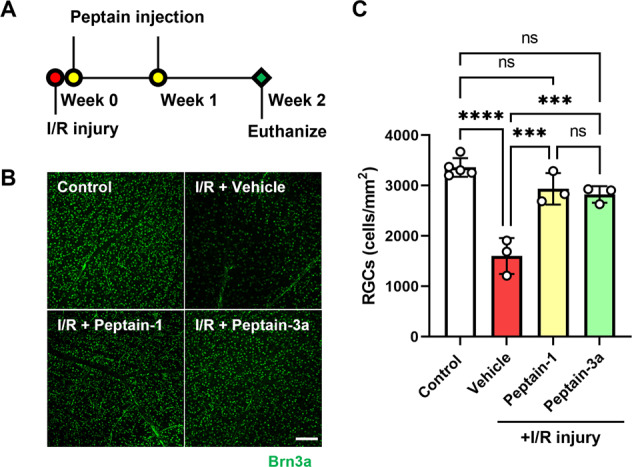
Peptains protect RGCs from ischemia/reperfusion (I/R) injury in mice. **A** Peptain injection timeline for I/R injury is shown. Mice were subjected to retinal I/R injury. Peptains (1 μl, 1 mg/mL) were injected into the vitreous humor immediately or one week after I/R injury. The vehicle-control group (I/R) was injected with 1 μl PBS (with 0.1% DMSO). **B** Fourteen days after injury, retinal flatmounts were prepared and the RGCs (green) were immunolabeled with a Brn3a antibody. **C** The bar graph shows the number of RGCs/mm^2^ in the mid-peripheral retina. The bars represent the mean ± SD of three independent experiments. Each data point represents one animal. ***p < 0.001, ****p < 0.0001, ns = not significant. Control = contralateral uninjured eye. Scale bar = 100 μm.

Retinal I/R injury leads to activation of the immune response and contributes to cellular damage, similar to the pathogenesis of glaucoma and diabetic retinopathy [42]. To determine the neuroprotective effect of peptains in vivo, mice were subjected to I/R injury. To achieve maximal protection, peptain-1 and peptain-3a were intravitreally injected with the highest soluble concentration (1 μl of a 1 mg/mL stock with 0.1% DMSO in PBS). Fourteen days after the I/R injury, the retinal flatmounts were immunostained with a brain-specific homeobox/POU domain protein 3A (Brn3a) antibody as a marker of RGCs. In vehicle-treated mice subjected to I/R injury, the number of Brn3a-positive RGCs in the mid-peripheral region of the retina was significantly decreased by 52% compared to that in uninjured control eyes (*p* < 0.0001; Fig. 2). However, intravitreal administration of peptain-1 and peptain-3a significantly (*p* < 0.001) inhibited RGC loss; the losses were only 13% and 16%, respectively, compared to the vehicle-treated eyes. There were no significant differences between the peptain-treated and control eyes. Together, these results suggested that peptain-1 and peptain-3a are highly effective in preventing RGC loss following I/R injury.

### Peptains inhibit RGC death in microbead (MB)-induced ocular hypertension in mice

**Fig. 3:**
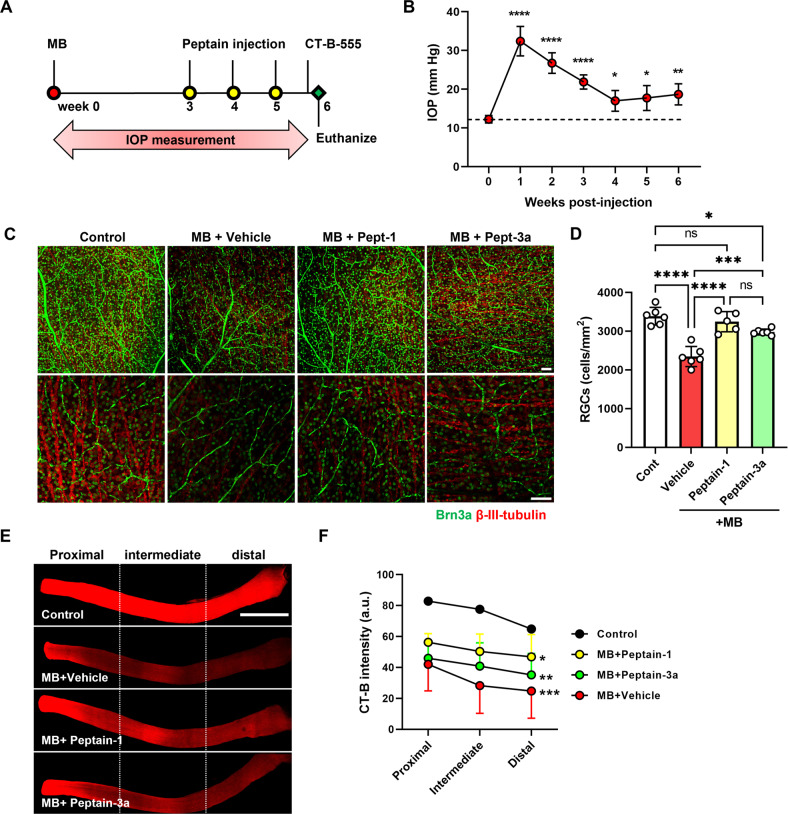
Peptains inhibit RGC death against microbead-induced ocular hypertension in mice. **A** Peptain injection timeline for a mouse model of glaucoma is shown. Two microliters of MB (from a stock of 5 × 10^6^ beads/mL) was injected into the anterior chamber. After 3 weeks, 1 µl of peptain-1 or peptain-3a was injected intravitreally at 1 µg/µl in PBS (with 0.1% DMSO) once a week for 3 weeks. Mice were injected intravitreally with CT-B one day before euthanasia. **B** IOP was monitored weekly during the 6-week follow-up period. **C** At 6 weeks post-MB injection, retinal flatmounts were immunostained with Brn3a (green) and βIII-tubulin (red) antibodies as markers for RGCs. Confocal microscopic images were captured from the mid-peripheral retina. Scale bar = 50 μm. **D** The bar graph shows the quantification of Brn3a-positive RGC loss in retinal flatmounts. Each bar represents the mean ± SD of 5–6 independent experiments. Each data point represents one animal. **E** Confocal images show the anterograde transportation of CT-B along the entire length of the optic nerve to the chiasm. **F** The CT-B intensity was quantified in proximal (close to the optic nerve head), intermediate, and distal (close to the optic chiasm) regions of the optic nerve. Scale bar = 1 mm. Data represent the mean ± SD of 5–6 independent experiments. *p < 0.05, **p < 0.01, ***p < 0.001, ****p < 0.0001, ns = not significant. Control = contralateral uninjured eye.

Next, we determined whether peptains were able to protect RGCs in a mouse model of glaucoma. The MB occlusion model has been used to impede aqueous outflow and elevate IOP in rodents [43, 44], rabbits [45] and a nonhuman primates [46]. Therefore, we injected MB into the anterior chamber and elevated IOP, as previously described [47]. Three weeks after MB injection, we intravitreally delivered either peptain-1 or peptain-3a (1 µg in 1 µl) once a week for 3 weeks (Fig. 3A). Upon MB injection, the IOP was significantly elevated within a week (*p* < 0.0001), after which it gradually declined over the following weeks of the six-week period but remained significantly (*p* < 0.05) above the control levels (Fig. 3B). The retinal flatmounts were immunostained with Brn3a and βIII-tubulin antibodies as markers for RGCs. MB injection caused ~31% RGC loss (Brn3a positive) relative to the contralateral control eyes. However, peptain-1 and peptain-3a significantly reduced RGC loss; the losses were only 4% (*p* < 0.0001) and 12% (*p* < 0.001), respectively (Fig. 3C, D). The RGC numbers were statistically insignificant in the peptain-1-treated eyes compared to the control eyes but were significantly (*p* < 0.05) lower in the peptain-3a-treated eyes, suggesting that peptain-1 was better than peptain-3a in protecting RGCs in this animal model of glaucoma.

### Peptains ameliorate axonal transport deficits in MB-induced ocular hypertension in mice

In separate experiments, we assessed axonal transport in RGCs using cholera toxin subunit-B (CT-B) as a neuronal tracer. Six weeks after MB injection, CT-B intensity was significantly (*p* < 0.001) reduced in the proximal (51%), intermediate (36%), and distal (38%) regions of the optic nerve in the vehicle-treated eyes compared to the uninjured control eyes (Fig. 3E, F). In the peptain-1-injected eyes, the axonal transportation deficits were mitigated; the CT-B intensities were 68%, 65%, and 72% of that of the uninjured control eyes at the proximal, intermediate, and distal regions, respectively. Peptain-3a (Fig. 3E, F) was slightly inferior to peptain-1 in its ability to prevent axonal transportation deficits but was still effective, as evidenced by the CT-B fluorescence intensities of 55%, 53%, and 54% of the uninjured control eyes at the proximal, intermediate, and distal regions, respectively. These results indicated that in the context of the clinical setting, in which glaucoma patients often have lost some degree of vision due to RGC loss, peptains are likely to provide protection against further loss of RGCs and optic nerve damage, perhaps halting or reducing continued vision loss.

### Peptains inhibit glial activation in MB-induced ocular hypertension in mice

**Fig. 4:**
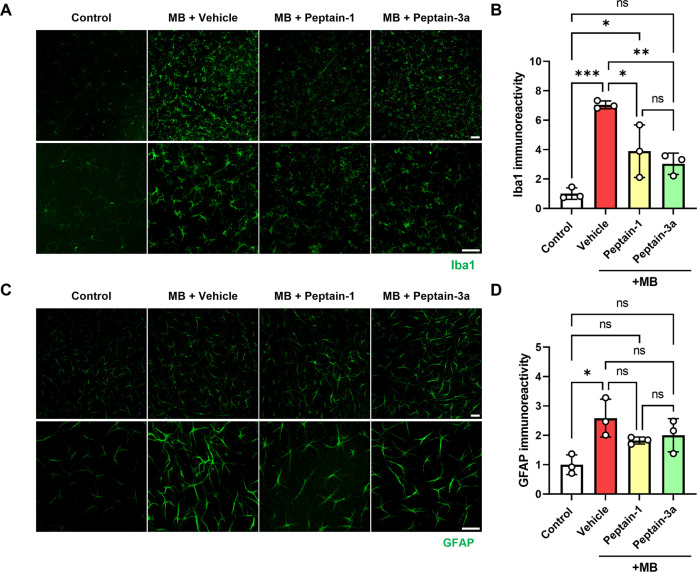
Peptains inhibit glial activation in MB-induced ocular hypertension in mice. The induction of ocular hypertension was performed as described in Fig. 3. MB was injected into the anterior chamber, and after 3 weeks, peptain-1 or peptain-3a was injected intravitreally at 1 µg/µl in PBS (with 0.1% DMSO) and subsequently once a week for 3 weeks. Six weeks after MB injection, retinal flatmounts were immunostained for Iba1 (**A**, **B**) and GFAP (**C**, **D**) to identify activated microglia and astrocytes. The bar graph **B** shows the fold change (mean ± SD) of Iba1 positive cells and **D** shows the fold change (mean ± SD) of GFAP fluorescence intensity in the ganglion cell layer. Each data point represents one animal. Scale bar = 100 μm.

In the pathogenesis of glaucoma, neuroinflammation occurs through activation of the residential glia [48, 49]. Previous studies have shown that highly activated microglia can release inflammatory cytokines, leading to inflammatory damage and synapse loss, which can contribute to the death of RGCs [50]. In addition, activation of astrocytes and Müller cells increases the expression of glial fibrillary acidic protein (GFAP) in response to injury, which is associated with detrimental effects on axonal transport [51]. Hence, we determined whether peptains suppress glial activation in an ocular hypertension mouse model. MB-mediated ocular hypertension for six weeks caused robust activation of microglia (Fig. 4A, B, *p* < 0.001) and astrocytes (Fig. 4C, D, *p* < 0.05). Peptain-1 (*p* < 0.05) and peptain-3a (*p* < 0.01) injections resulted in significantly lower numbers of ionized calcium-binding adapter molecule 1 (Iba1)-positive microglia (Fig. 4B). The GFAP immunoreactivity was slightly lower in the peptain treated than vehicle-treated retinas (Fig. 4C, D). These results suggested that intravitreal delivery of peptains can suppress glial activation in glaucoma.

### Peptains inhibit RGC death in silicone oil (SO)-induced ocular hypertension in mice

**Fig. 5:**
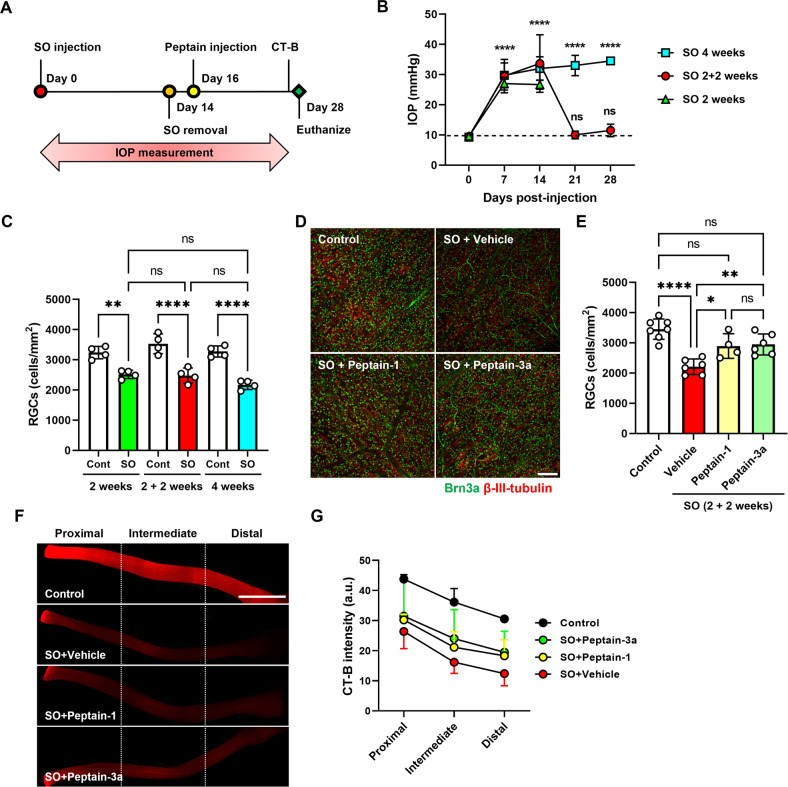
Protective effects of peptains against RGC death in SO-induced ocular hypertension. **A** The SO and peptain injection timeline is shown. Mice were injected with silicone oil (SO, 2 µl, 1000 mPa.s) into the anterior chamber until the oil covered the iris; after 2 weeks, the oil was removed. Peptain-1 or peptain-3a was intravitreally injected at 1 µg in 1 µl of PBS (with 0.1% DMSO) 2 days after SO removal. **B** The IOP was monitored once a week during the 28-day follow-up period. The injection of SO elevated the IOP, and its removal returned IOP to the normal range. **C** Mice were euthanized, and the retinal flatmounts were immunostained with Brn3a at 2 weeks post-SO treatment, at 2 weeks post-SO treatment + 2 weeks post-SO removal, (2 + 2 weeks), and as another control, we injected SO, and mice were euthanized after 4 weeks without removal (4 weeks). The bar graph shows the quantification of Brn3a-positive RGCs in retinal flatmounts. *N* = 4. **D** Two weeks after SO removal (2 + 2 weeks), retinal flatmounts were immunostained for Brn3a (green) and βIII-tubulin (red) antibodies. Confocal microscopic images were captured from the mid-peripheral retina. Scale bar = 100 μm. **E** The bar graph shows the quantification of Brn3a+ RGCs in retinal flatmounts. Each data point represents one animal. **F** Confocal images showed the anterograde transportation of CT-B along the entire length of the optic nerve to the chiasm. **G** The CT-B intensity was quantified in the proximal, intermediate, and distal regions of the optic nerve. Scale bar = 1 mm. Data represent the mean ± SD of 4–6 independent experiments. *p < 0.05, **p < 0.01, ****p < 0.0001. ns = not significant. Control = contralateral uninjured eye.

Previous studies have shown that SO-induced ocular hypertension mimics the pathology of acute angle-closure glaucoma [52, 53]. The intracamerally injected SO leads to pupillary blockade, which impedes aqueous outflow, resulting in the retention of aqueous humor in the posterior chamber and consequently increasing IOP. One distinct advantage of this model is that elevated IOP can be reversed to basal levels by removing SO, and therefore, this model is useful to evaluate neurodegeneration in the absence of elevated IOP, similar to situations in some glaucoma patients who show continued neurodegeneration after being treated with IOP-lowering drugs. Two weeks after SO injection, the oil was removed from the anterior chamber. Two days after SO removal, peptain-1 or peptain-3a (1 µg in 1 µl PBS) was injected into the vitreous humor (Fig. 5A). The SO injection significantly elevated the IOP after 1 week, and it remained elevated for 4 weeks post-SO injection when compared to the uninjected eyes (*p* < 0.0001; Fig. 5B). The IOP dropped to normal levels after the removal of SO. Mice were euthanized 2 (SO 2 weeks) or 4 weeks (SO 4 weeks) after SO injection or 2 weeks after SO removal (SO 2 + 2 weeks). The retinal flatmounts were immunostained for the RGC marker Brn3a. The SO injection caused 22% and 34% RGC loss relative to uninjected contralateral eyes after 2 and 4 weeks, respectively (Figs. 5C and S3). In the eyes in which SO was removed after 2 weeks, we observed a 30% loss when compared to uninjected contralateral eyes. This corresponded to an increase of 8% loss compared to RGC numbers at 2 weeks. These results supported the idea that RGCs continue to die after normalizing IOP. We next assessed the effects of peptains (administered post IOP normalization) on RGC survival. When the vehicle-treated eyes were compared to the control eyes, 64% of RGCs were observed (Fig. 5D, E). However, the remaining RGCs were 84% and 85% in the peptain-1 and peptain-3a treated eyes. These values were significantly higher when compared to the vehicle-treated eyes (*p* < 0.05 vs peptain-1 and *p* < 0.01 vs peptain-3a treated eyes). These results suggested that the peptains protected RGCs during the 2-week period of normal IOP after a 2-week period of elevated IOP.

### Peptains improve axonal transport deficits in SO-induced ocular hypertension in mice

Similar to the MB experiment, we tested whether treatment with peptains prevented axonal transport deficits after SO removal. In the vehicle-treated eyes, the fluorescence intensity of CT-B in the optic nerve was drastically decreased in the proximal, intermediate, and distal regions (60%, 45%, and 41% of control in the same regions, respectively; Fig. 5F, G). Upon peptain injection (2 days after SO removal), axonal transportation was preserved compared to vehicle-treated eyes. Values of 69%, 59%, and 60% in the peptain-1 -treated eyes and 72%, 66%, and 64% of control CT-B fluorescence intensities in the peptain-3a eyes were observed in the proximal, intermediate and distal regions of the optic nerve, respectively. These results indicated that SO-mediated IOP elevation causes axonal damage, which was inhibited by peptains when administered two weeks postinjury.

### Peptains inhibit glial activation in SO-induced ocular hypertension in mice

**Fig. 6:**
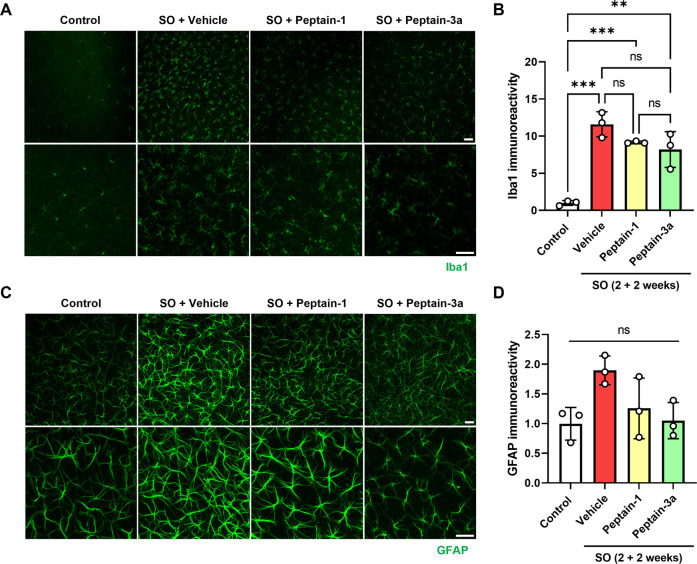
Peptains inhibit glial activation in SO-induced ocular hypertension. The induction of SO-mediated ocular hypertension was performed as described in Fig. 5. SO was injected into the anterior chamber, and after 2 weeks, the oil was removed. Peptain-1 or peptain-3a was intravitreally injected at 1 µg in 1 µl of PBS (with 0.1% DMSO) 2 days after SO removal. Four weeks after injury, retinal flatmounts were immunostained with Iba1 (**A**, **B**) and GFAP (**C**, **D**) antibodies to identify activated microglia and astrocytes. The bar graph **B** shows the fold change (mean ± SD) of Iba1 positive cells and **D** shows the fold change (mean ± SD) of GFAP fluorescence intensity in the ganglion cell layer. Each data point represents one animal. Scale bar = 100 μm.

We assessed the effects of peptains on glial activation in SO-induced ocular hypertension. In the SO removal groups, the vehicle-treated eyes showed microglial and astrocyte activation, as evidenced by an increase in staining for Iba-1 and GFAP, especially in the ganglion cell layer (Fig. 6). The peptain-treated groups showed slightly lower numbers of activated microglia (Fig. 6A, B), and GFAP immunoreactivity (Fig. 6C, D), suggesting that peptains are capable of reducing neuroinflammation in SO-induced glaucoma.

## Discussion

The objectives of this study were to evaluate the efficacy of peptains in preventing RGC death caused by deprivation of neurotrophic factors and to determine whether intravitreally injected peptains can prevent RGC death in animal models of glaucoma. Reduced levels of neurotrophins are believed to cause RGC loss in glaucoma [54]. Our results showed that peptains inhibited RGC death due to neurotrophic factor deprivation. Our results also demonstrated that intravitreal injection of peptains significantly reduced RGC death despite elevated IOP in I/R injury and two animal models of glaucoma. It is tempting to propose that the inhibition of RGC death occurred as a result of the interception of key apoptotic steps by peptain-1, given its established antiapoptotic activity [36, 37, 55] and the fact that RGCs die of apoptosis in animal models and human glaucomatous eyes [56, 57]. Mechanistically, peptain-3a is likely to be similar to peptain-1, given that it exhibits considerable sequence homology with peptain-1 and that the two are derived from members of the small heat shock protein family. However, to gain better insights, the biochemical steps intercepted by peptains during the rescue of RGCs must be delineated in future studies.

Our results from this and a previous study suggested that intravitreally injected peptain-1 likely entered RGCs before it prevented cell death. In our previous study with peptain-1, we demonstrated its spontaneous entry into cultured RGCs [29], and in the current study, the addition of peptain-1 to cultured RGCs inhibited their apoptosis, suggesting its cell entry. Furthermore, we have previously demonstrated that peptain-1 penetrates lens epithelial cells on its own when added to the culture medium [37]. Peptain-1 has also been shown to restore mitochondrial glutathione and to prevent apoptosis mediated by oxidative damage in retinal pigment epithelial cells [58]. Thus, it is conceivable that intravitreally injected peptain-1 entered RGCs and protected them in our animal models. Similar to peptain-1, peptain-3a enters cells on its own [38]. We have previously shown that the C-terminal amino acid sequences LKVK in peptain-1 and IAVK in peptain-3a are obligatory for their cell entry [38]. It is possible that peptains gained entry into RGCs through the aid of these sequences. A cell surface amino acid transporter capable of transporting peptain-1 has been identified in retinal pigment epithelial cells [36]. Whether a similar transporter carried peptains into RGCs remains to be determined. The proximity of RGCs to the vitreous humor may further enable the entry of peptains into RGCs.

The ability of peptains to protect RGC axonal transport is significant, given that axonal injury is a key determinant of vision loss in glaucoma [59]. Axonal injury is believed to be caused by multiple factors, including neurotrophic factor deprivation, inflammation, and damage to the cytoskeleton. Whether peptains prevented these abnormalities in our animal models is not known at this point and will require additional work. However, we can speculate that peptains could have protected the RGC cytoskeleton, as sHsps have been shown to protect the cytoskeleton in stressed cells [60, 61]. Additionally, RGC axons and the optic nerve head contain high densities of mitochondria [62], and RGC mitochondrial damage is frequently observed in animal models of glaucoma [62, 63]. Peptains may have prevented mitochondrial damage. Supporting this view is our previous observation that peptain-1 restores retinal mitochondrial cytochrome c oxidase subunit 6b2 levels in a rodent model of glaucoma [29]. Furthermore, sHsps, including HspB5, are known to reduce mitochondrial abnormalities in stressed cells [64].

Inflammation through glial activation promotes RGC death in animal models of glaucoma [9]. We found that peptains reduced glial activation in both the SO and MB ocular hypertensive models, which suggested that they decreased the production of inflammatory cytokines by the activated glia. Further studies are necessary to determine the effects of peptains on retinal inflammatory cytokine levels in eyes with elevated IOP.

The SO model permitted us to remove the SO after a 2-week period of elevated IOP, resulting in the normalization of the IOP. This allowed us to test whether the injected peptains protected RGCs after a brief 2-week elevation in IOP. While SO removal after two weeks drastically reduced the IOP, RGC death continued to progress over the ensuing two-week period (from 22% at 2 weeks to 30% at 2 + 2 weeks). Our results clearly showed that the two peptides were able to protect more than 84–85% of RGCs and that both peptides reduced the defects in anterograde transport through the optic nerve. An interesting observation here is that the peptain treatment two weeks after SO treatment and subsequent removal led to the detection of more Brn3a-positive RGCs after an additional 2-week post-SO recovery period than at the time point before peptain treatment (2 weeks post-SO injection, Fig. S3). We interpret these results as peptains increasing the expression of Brn3a in RGCs that were on the brink of death, thereby rescuing them. Mice possess more than 40 subtypes of RGCs, and most RGCs [65] express Brn3a. However, it is possible that the RGCs that did not normally express Brn3a expressed Brn3a as a result of peptain treatment; further work is needed to determine the effects of peptains on the expression of Brn3a. Nonetheless, we view our findings on the SO model as significant because the experiment somewhat recapitulated the situations observed in glaucoma patients.

A limitation of this study is that we tested peptains at one concentration; we tested the ability of peptains at their maximum soluble concentrations, anticipating that we would achieve the highest effects. Therefore, we cannot rule out the possibility that the peptain concentrations were more than what was required to obtain the highest protection of RGCs. A dose-response experiment to determine the minimum effective concentration is needed in a future study.

In conclusion, even though IOP lowering reduces functional deficits in RGCs [66] and generally prevents further damage to vision [67], ~20% of patients still show disease progression despite decreased IOP [68], possibly due to the death of RGCs from mechanisms that are poorly understood. Peptain therapy might be most beneficial to those patients who do not respond to IOP-lowering therapies. This option may also be advanced as an adjunct therapy to IOP-lowering therapies to prevent RGC death to save vision.

## Materials and methods

### Peptides

Peptain-1 and peptain-3a were obtained from Peptide 2.0 (>95% pure, Chantilly, VA). All peptides used in this study were confirmed by mass spectrometry to have the expected molecular weights. For in vivo studies, peptains were dissolved in a small amount of DMSO and then diluted in PBS to the desired concentration. The final concentration of DMSO was <0.1%. For in vitro tests, peptains were dissolved in PBS.

### Chaperone activity

The chaperone activity of peptain-1 and peptain-3a was assessed using insulin (Cat# I5500, Sigma–Aldrich, St. Louis, MO, USA) and citrate synthase (Cat# C3260, Sigma–Aldrich) as client proteins, as previously described [38]. Insulin (0.3 mg/mL) and dithiothreitol (20 mM, Cat# D9163, Sigma–Aldrich) were incubated in 50 mM phosphate buffer, pH 7.4, at 25 °C for 1 h in the presence or absence of peptain-1 (20–80 μM) or peptain-3a (0.07–8.7 μM). Citrate synthase (0.167 mg/mL) was incubated in 10 mM HEPES buffer, pH 7.4, at 43 °C for 1 h in the presence or absence of peptain-1 or peptain-3a. The assay volume in each case was 200 μl. The kinetic profile of aggregation was monitored by measuring light scattering at 360 nm in a 96-well microplate reader (SpectraMax 190, Molecular Devices, Sunnyvale, CA).

### Primary RGC apoptosis assay

The isolation of rat primary RGCs from postnatal day 4–6 Sprague–Dawley pups was performed according to a previously published protocol [69]. The purity of the culture was found to be between 90–95%. RGCs were seeded onto glass coverslips, and after 5 days of culture, cells were incubated with vehicle (DPBS), peptain-1 (12.5 μg/mL), or peptain-3a (1.25 μg/mL) in either DMEM with high glucose (no trophic factors) or in full RGC (Sato) media (with trophic factors). After 48 h of incubation at 37 °C with 10% CO_2_, the Image-iT LIVE Green Caspase-3 and -7 Detection Kit (Cat# I35106, Invitrogen) was used to stain cells with FLICA (labeling caspase-3 and -7 in apoptotic cells) and with Hoechst, labeling all cells, and propidium iodide (PI, labeling dead cells). Briefly, RGCs were incubated with 1x FLICA for 60 min followed by Hoechst/PI for 5 min. The cells were then washed and imaged using a Cytation5 Cell Imaging Multimode Reader (Agilent, Santa Clara, CA). Manual cell counting was performed using ImageJ software for Hoechst-positive cells with 0.3–1.0 circularity, which passed the threshold (determined by the level of background to ensure that only true positives were counted). Cells costained with PI and FLICA were categorized into cells undergoing apoptosis. Cells labeled only with PI were marked as dead. The experiment was repeated three times. The data are presented as the percentages of apoptotic and dead cells compared to controls (vehicle-treated group).

### Animals

All animal experiments were reviewed and approved by the University of Colorado and North Texas Eye Research Institute, UNT Health Science Center’s Institutional Animal Care and Use Committee and performed under adherence to the ARVO Statement for the Use of Animals in Ophthalmic and Vision Research. Both male and female wild-type mice were obtained from Jackson Laboratories (C57BL/6 J, Stock No: 000664, Bar Harbor, ME, USA) or bred in-house with a 12:12-h light/dark cycle with ad libitum food and water. Twelve to 13 weeks old mice were randomly assigned one of three groups: vehicle, peptain-1 or peptain-3a.

### Retinal ischemia–reperfusion (I/R) injury

I/R injury was performed as previously described [70]. Briefly, mice were anesthetized with an intraperitoneal injection of ketamine/xylazine, eyes were anesthetized with topical 0.5% proparacaine hydrochloride ophthalmic solution (Akorn, Somerset, New Jersey), and anesthesia was confirmed by the toe-pinch pain test. The animals were then placed on a heating pad to maintain their body temperature throughout the procedure. The anterior chamber of the right eye of each mouse was cannulated with a 33-gauge needle connected to an elevated saline reservoir. The height of the reservoir was adjusted to achieve an IOP of 120 mm Hg. After 60 min, the needle was rapidly removed. Peptain-1 or peptain-3a (1 μl from 1 mg/mL stock) was intravitreally injected immediately after I/R injury and after 2 days. The control group was injected with 1 μl PBS containing 0.1% DMSO. The animals were euthanized on day 14 post- I/R injury.

### MB-induced ocular hypertensive mouse model

Mice were anesthetized as described above. Ocular hypertension was induced unilaterally by injection of polystyrene MB (10-μm diameter, FluoSpheres, Invitrogen, Carlsbad, CA) into the anterior chamber of the right eye of each animal, as previously described [71]. Briefly, MB was reformulated at a concentration of 5 × 10^6^ beads/mL in PBS. The cornea was gently punctured near the center using a 33 G needle, and a small air bubble was injected to deepen the anterior chamber. MB (2 μl) was injected into the anterior chamber under the bubble via a blunt 33G needle connected to a 10 μl Hamilton syringe. Antibiotic ophthalmic ointment (Akorn, Inc. Cat# AP704009, Lake Forest, IL) was applied topically to the injected eye to prevent infection. IOP was monitored once weekly for 6 weeks using the TonoLab tonometer (Colonial Medical Supply, Espoo, Finland). Three weeks after MB injection, peptain-1 or peptain-3a was injected intravitreally (1 µg in 1 µL PBS), and then subsequently once a week for 3 weeks.

Mice were considered to have ocular hypertension when the IOP was > 16 mmHg after 1 week of MB injection. Three mice that did not show elevated IOP after MB injection or showed vitreous hemorrhage were excluded from the study.

### Induction of IOP elevation by injection of SO

Mice were anesthetized as described above. SO-induced reversible ocular hypertension was induced as previously described [52]. Briefly, a 33G needle was inserted superotemporally into the anterior chamber without injuring the lens or iris. Through this puncture, ~2 μl SO (1000 mPa.s, Silikon, Alcon Laboratories, Fort Worth, Texas) was gradually injected intracamerally until the oil droplet expanded to cover most of the iris, and the needle was then slowly withdrawn. After the injection, ophthalmic antibiotic ointment was applied to the surface of the injected eye. After 2 weeks, the SO droplet was removed from the anterior chamber by aspiration using a 33G needle. PBS was then injected into the anterior chamber using a 33G needle attached to a 3 mL Luer lock syringe to allow SO outflow through an inferior tunnel incision. Antibiotic ophthalmic ointment was applied topically to the eye after SO removal. The IOP of the eye was monitored once weekly for 4 weeks after SO injection using the TonoLab tonometer under anesthesia (5% isoflurane at 2 L/min mixed with oxygen). Peptains were injected intravitreally 2 days after SO removal. Two mice that showed signs of anterior segment inflammation were excluded from the study.

### Immunostaining of mouse retinal flatmounts

The animals were euthanized at the indicated times, and the eyes were enucleated and fixed with 4% PFA overnight at 4 °C. The next day, the retinas were dissected out and washed extensively in PBS before blocking (5% normal donkey serum and 1% Triton X-100 in PBS) overnight. The retinal flatmounts were immunostained with Brn3a (1∶400 dilution, Cat# MAB1585, EMD Millipore, Bedford, MA) and βIII-tubulin antibodies (1:400 dilution, Cat# T8578, Sigma–Aldrich, St. Louis, MO) as markers for RGCs or Iba1 (1:400 dilution, Cat# ab178846, Abcam, Cambridge, MA) or GFAP (1:400 dilution, Cat# 12389, Cell Signaling Technology) antibodies as markers for activated microglia and activated astrocytes, respectively. After 3 days, Alexa Fluor 488-conjugated donkey anti-mouse (1:250 dilution, Cat# A-11001, Invitrogen), Alexa Fluor 488-conjugated donkey anti-rabbit (1:250 dilution, Cat# A-21206, Invitrogen), or Texas red-conjugated goat anti-rabbit IgG (1:250 dilution, Cat# T-2767, Invitrogen) antibody was incubated overnight at 4 °C. Four fields from mid-peripheral regions of the retina were imaged using a confocal microscope (Nikon ECLIPSE Ti, Japan). The percentage of RGC survival was calculated as the ratio of surviving RGC numbers (cells/mm^2^) in the injured eyes compared to the contralateral uninjured eyes.

### Axonal transportation

CT-B, a neuronal tracer, was used to assess anterograde axonal transport as previously described [29]. Mice were anesthetized and intravitreally injected with 1 μl of 0.2% CT-B (Alexa Fluor 555-conjugate, Thermo Fisher, Cat# C22843) a day before euthanasia. After 24 h, the mice were killed, and the optic nerves were dissected, fixed in 4% PFA overnight, and dehydrated in methanol for 5 min. The optic nerves were cleared by incubation with Visikol® HISTO-1™ for 1 week, then transferred to Visikol® HISTO-2™ and incubated for an additional 1 week. The optic nerves were imaged using confocal microscopy along the entire length to the optic chiasm and stitching with 15% overlap. The mean fluorescence intensities were calculated using ImageJ software (NIH).

### Statistical analysis

GraphPad Prism software version 9.2.0 (GraphPad Prism Software, Inc., San Diego, CA) was used for statistical analyses. We used one-way ANOVA and Tukey’s multiple comparisons tests to determine the significance of differences among the animal groups. The variance was similar between the groups and a *p* value of <0.05 was considered statistically significant. The investigators were not blinded during experiments and data analysis. Conclusions were made based on quantitative parameters.

## Supplementary information

Supplemental material

Reproducibility Checklist

## Data Availability

All study data are included in the main text and the supplementary information files.
