# Supplementary material for: Peptains block retinal ganglion cell death in animal models of ocular hypertension: implications for neuroprotection in glaucoma
Source: Cell Death Dis. 2022 Nov 15;13(11):958. doi: 10.1038/s41419-022-05407-2 (PMC9666629; doi:10.1038/s41419-022-05407-2)
Supplement: Supplementary file 1 — Supplemental material [file 41419_2022_5407_MOESM1_ESM.docx]

**
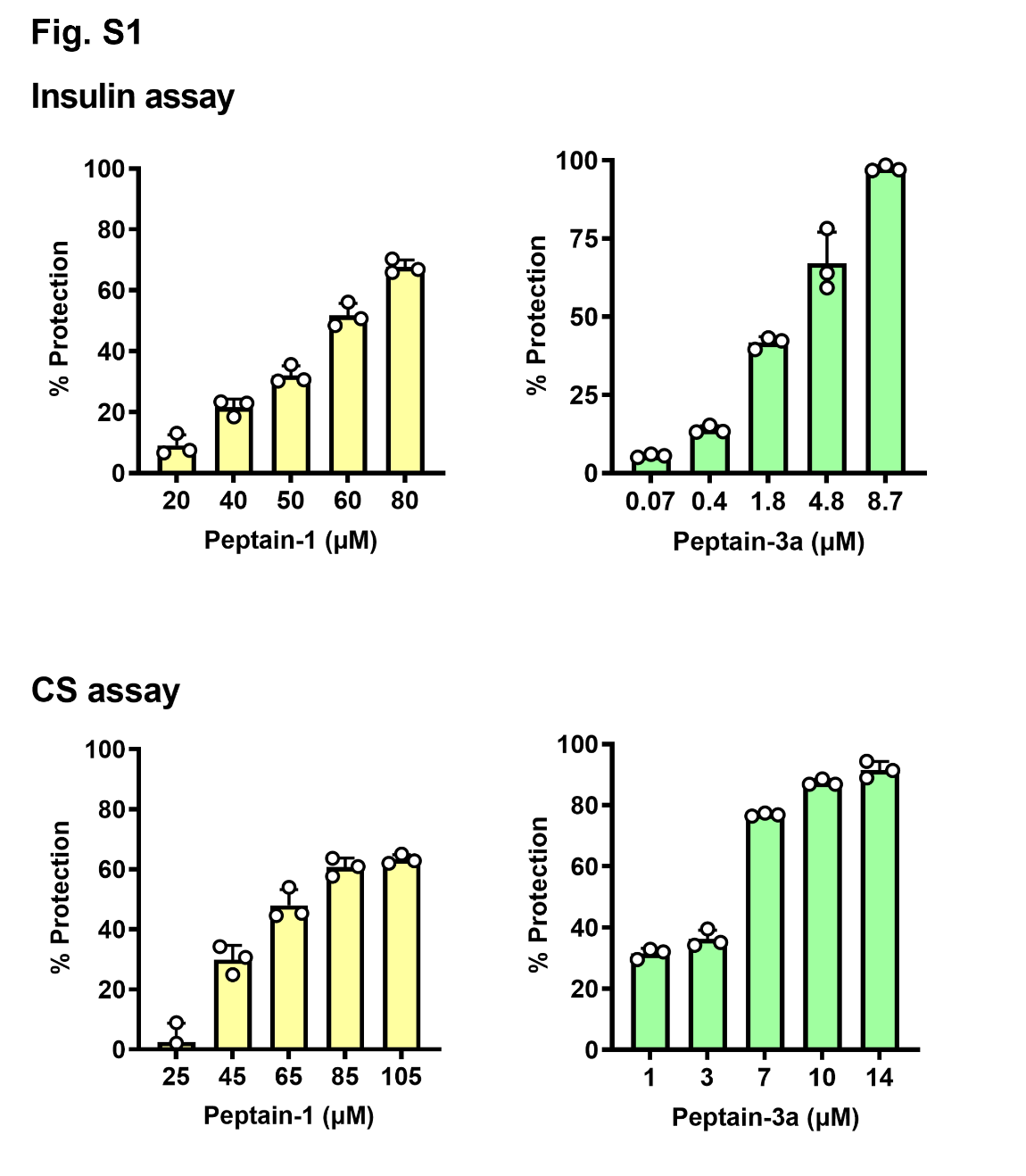
**

**Supplementary Figure 1**. Relative chaperone activity of peptains. Chaperone activity was assessed using insulin or citrate synthase (CS) as the client protein. The kinetic profile for insulin or CS aggregation was monitored by measuring scattering at 360 nm, and percent protection was calculated based on protection against client protein aggregation in the presence or absence of the peptains. The bar graphs represent the mean ± SD of 3 independent experiments.

**
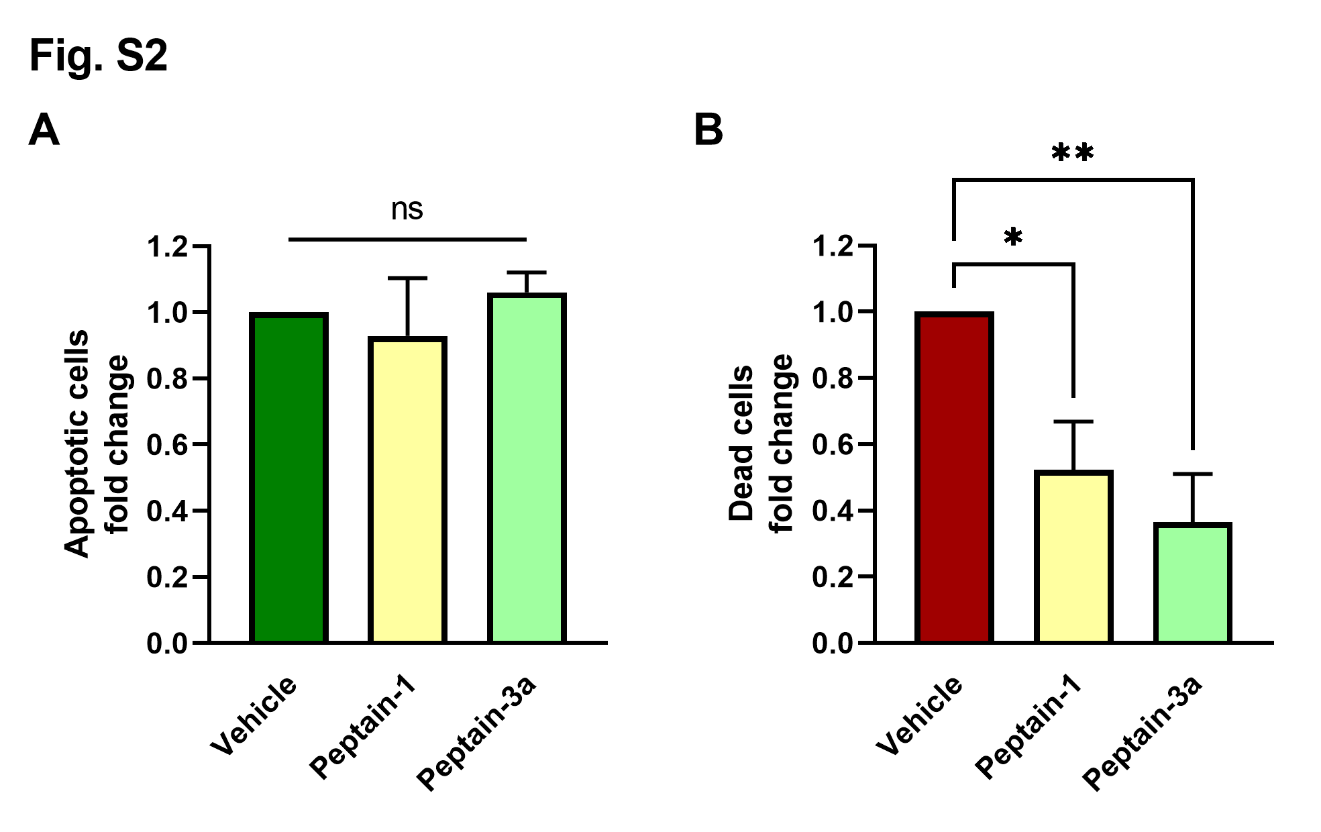
**

**Supplementary Figure 2.** Peptains do not cause RGC apoptosis (**A**) or death (**B**). Primary rat RGCs were isolated and cultured in the presence or absence of peptains (with TF) for 48 h. The bars represent the mean ± SD of three independent experiments. *p<0.05, **p<0.01, ns= not significant.

**
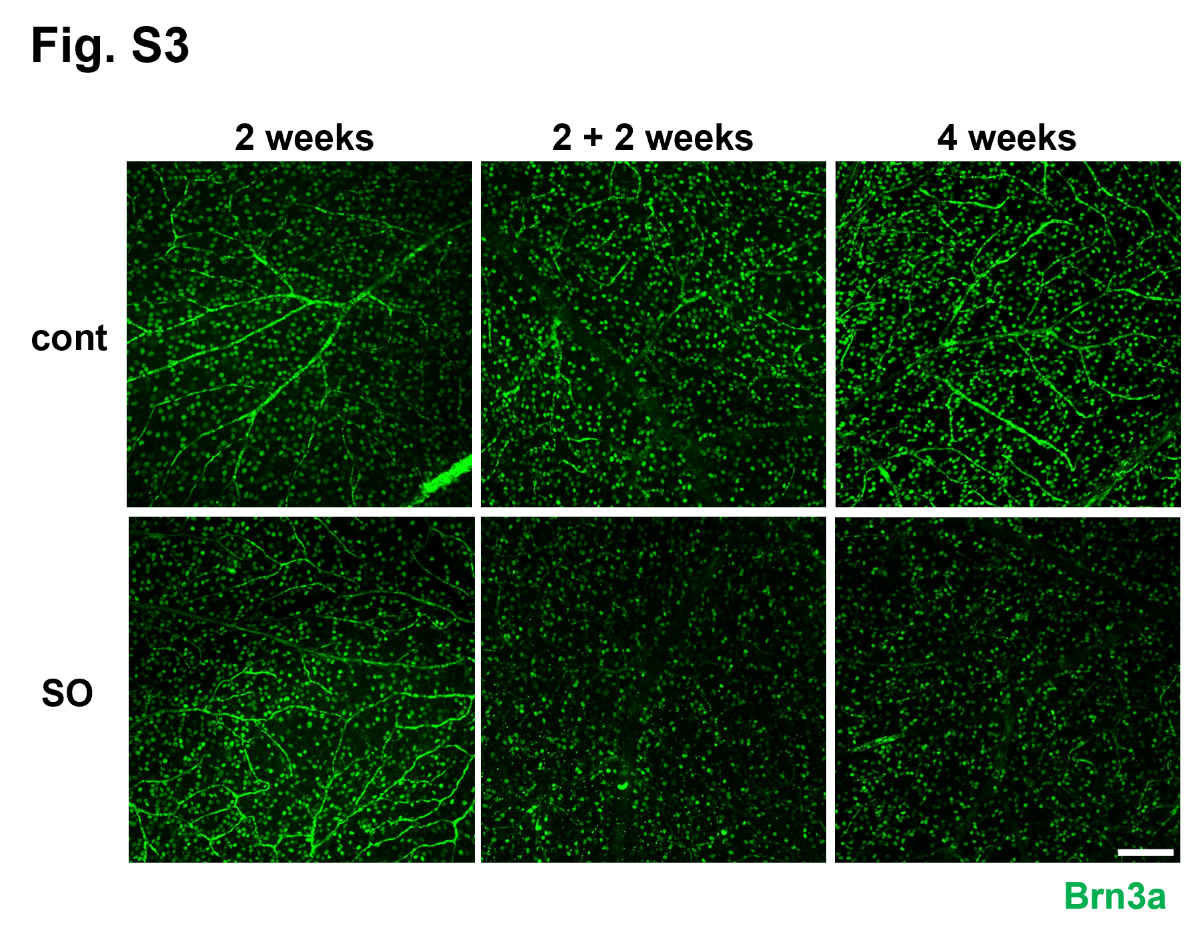
**

**Supplementary Figure 3**. The induction of SO-mediated ocular hypertension was performed as described in Fig. 5. SO was injected into the anterior chamber until the oil covered the iris. After 2 weeks, the mice were euthanized (2 weeks), or the oil was removed. Two weeks after SO removal, the mice were euthanized (2 + 2 weeks). In some mice, SO was not removed; they were euthanized after 4 weeks (4 weeks). Whole-mount retinas were immunostained with Brn3a, and confocal microscopic images were captured from the mid-peripheral retina. cont = uninjured contralateral eyes. Scale bar = 100 μm.
